# Lake shape and the characteristics of migration behavior modify Atlantic salmon smolt migration success through lakes

**DOI:** 10.1111/jfb.15972

**Published:** 2024-10-23

**Authors:** Matthew Newton, Joseph McCallum, Hannele M. Honkanen, Alastair Stephen, Jessie M. Lilly, Danielle L. Orrell, Amy Green, Louise Chavarie, Jessica R. Rodger, Colin E. Adams

**Affiliations:** ^1^ Scottish Centre for Ecology and the Natural Environment, School of Biodiversity One Health and Veterinary Medicine University of Glasgow Glasgow UK; ^2^ Scottish & Southern Energy Perth UK; ^3^ Atlantic Salmon Trust, The Walled Garden, Kilgraston Perth UK

**Keywords:** environmental cues, migration pathways, simulated migration

## Abstract

Migration is a high‐risk behavior. For the Atlantic salmon, *Salmo salar*, migrating from its river nursery area to marine feeding grounds, the magnitude of risk varies with habitat type. Passage through lakes, in particular, is associated with low rates of migration success. Downstream migrating salmon smolts are rheotactic when migrating in rivers, but lakes typically provide poorer directional currents for migrating salmon. In this study we tested if, in the absence of clear navigational cues in lakes, Atlantic salmon smolts switch to a random search strategy to find the outflowing river. We constructed random search simulations to test if lake basin shape has an effect on migration success. We also compared simulated migration characteristics with migrations of salmon smolts through five real lakes for which there are telemetry data for migrating salmon. Correlated random walk simulations showed that a random search strategy could be successful for all lake shapes tested but was more successful in curved (round and elliptical) than rectangular basin shapes. Rectangular basins with the migration start and stop points at the ends of the lake had a higher success than those where these points were perpendicular to the axis of the lake. In general, a random walk model predicted the migration success rate of fish tracked through real lakes. However, for two lakes the simulated migration success exceeded that of actual success, suggesting that fish passing through these lakes were not adopting a random search strategy. We speculate that this is the result of either conflicting navigational cues which inhibit smolts from finding the lake outlet or that they temporarily suspend migration (e.g., to feed). Modelling predicted that for small lakes, directional swimming in short steps (ca. 100 m) followed by turns with very low variation from the direction of travel resulted in the highest migration success. For larger lakes, longer step lengths but also with low turn variation (simulated turning angle drawn from distributions of standard deviation 2**°** and 5**°** around a mean of 0**°**) resulted in the highest migration success. We conclude that navigation in downstream migrating salmon smolts switches from rheotaxis in rivers to a random search tactic in lakes except where residual flow cues in some lakes prevent this, at times resulting in suboptimal navigation outcomes.

## 1 INTRODUCTION

Despite being an activity that carries a very high level of risk (Alerstam et al., 2003; Mangel & Satterthwaite, 2008), long‐distance migration has evolved on multiple occasions in multiple lineages (Cresswell et al., 2011). Migration should only evolve and be maintained in a species when the fitness benefits of migrating outweigh its costs (Adams et al., 2022; Mangel & Satterthwaite, 2008). The costs of migration are manyfold, complex, and poorly quantified for most species (see Alerstam et al., 2003 for a review). The energetic expenditure for the individual making the migration journey is one obvious cost (Bonte et al., 2012). In addition migrating to a new habitat can increase an animal's exposure to novel predators, diseases, and parasites (Cresswell et al., 2011; Dingle, 1996; Furey et al., 2015). One nearly ubiquitous risk for migrating individuals of most species is the risk of navigational error during migration. If this occurs, then the migrant may fail to reach the intended location and ultimately this may result in mortality.

The Atlantic salmon, *Salmo salar* L., typically makes a long‐distance migration from its spawning and juvenile nursery areas in freshwater rivers to the open ocean (Thorstad et al., 2011). Migration for this species results in higher fitness, acquired through greater feeding opportunity, which leads to faster growth, larger body size, and enhanced reproductive output for both females (Sandlund et al., 2014) and males (Hutchings & Myers, 1994). However, migration for Atlantic salmon can be costly. For example, Thorstad et al. (2012) reviewed the literature on migration behavior for this species in rivers, estuaries, and inshore coastal marine habitats. They found median mortality for migrating Atlantic salmon smolts in rivers, estuaries, and coastal areas to be 2.3, 6, and 1.4% km^−1^, respectively. However, it is important to note that variation around these medians was high. Existing literature points towards marked between‐study and between‐habitat variation in migration success, superimposed on a general pattern of high levels of loss in Atlantic salmon smolts as they migrate to sea from freshwater.

One habitat type that was not examined by Thorstad et al. (2012) was freshwater standing waters, such as lakes and reservoirs. However, a number of studies have now shown that migration success for Atlantic salmon smolts though lake systems is low. For example, in a study of the Imsa catchment, Norway, migration losses through two lakes (Storavatnet and Kyllestadvatnet) ranged between 96% and 52%. Honkanen et al. (2021) showed migration losses ranging between 16 and 53% km^−1^ amongst three lakes in northwest Scotland. Additionally, an estimated 43% of Atlantic salmon smolts were lost during migration through Loch Lomond, in west‐central Scotland (Lilly et al., 2022). Two studies on migration success of salmon smolts through a Danish reservoir showed consistent migration success of around only 10% (Aarestrup et al., 1999; Jepsen et al., 1998). Lastly, a study in an Irish lake revealed a ca. 25% migration success rate for Atlantic salmon smolts through Lough Earn (Kennedy et al., 2018).

Several recent studies have suggested that the low migration success through standing fresh waters may be due to smolts not taking the most efficient route through the lakes, instead frequently taking highly tortuous routes to reach the outflowing river. This behavior has been shown to markedly extend their total migration distance and migration time, and thus their migration costs (Honkanen et al., 2018, 2021; Huusko et al., 2017; Lilly et al., 2022). For example, 28 Atlantic salmon smolts that successfully migrated through Loch Lomond covered a mean distance of 56 km (direct distance is 8.8 km), with one fish that successfully migrated taking a pathway of at least 246 km in length (Lilly et al., 2022). In a study of the in‐lake migration movements of Atlantic salmon smolts migrating through three lakes in northeast Scotland, 46%–52% of all recorded movements were in the opposite direction to the most direct route to the outflowing river (Honkanen et al., 2021).

A number of studies have indicated that low migration success through standing freshwaters is the result of a lack of reliable navigational cues, where the extended migration time is likely to result in greater energy expenditure and may extend a period of exposure to predators (Lennox et al., 2021). Aarestrup et al. (1999) suggested that salmon smolts may need to actively swim to find the lake outlet. Hanssen (2020) has shown that the movement patterns of salmon smolts through Lake Evangervatnet, Norway more closely resemble random search movements than navigation‐directed migration. In Loch Lomond, Scotland, characteristics of salmon smolt migration behavior closely resembled the output of a random walk model at least until fish entered an area close to the outlet river (the Goldilocks Zone), where there was evidence of more directed pathway migration (Lilly et al., 2022).

If salmon smolts are using random search behavior to migrate through freshwater lakes, then it is plausible that characteristics of the lake might significantly influence the success of such behavior. Thus, some lake shapes may result in higher migration success than others. Equally, some patterns of behavioral search patterns are likely to be more effective than others.

In the study reported here, we constructed a series of correlated random walk models (Hanssen, 2020) to test the effect of lake shape on the migration success of simulated salmon smolt migrations. In addition, we examined two elements of search behavior that are likely to affect migration success: (a) the angle at which a smolt makes a turn to swim in a different direction and (b) the distance that a smolt may swim in a straight line before initiating a turn (the step‐length). We also constructed random walk models for smolts migrating through six natural lakes for which we have empirical data on Atlantic salmon smolt migration and compared the characteristics of actual migration behavior with those of modelled migration simulations.

## 2 METHODS

### 2.1 Correlated random walk model

Simulated smolt migration pathways were generated in the form of correlated random walks using the ‘crw_in_polygon()’ function in the glatos package (Holbrook et al., 2021) in R V4.0.3 (R Core Team, 2020). Simulated pathways comprised a series of equal‐length, fixed‐vector migration steps with the turning angle between each step drawn from a Gaussian distribution of turning angles. To account for the general tendency of animals to move forward, the direction of travel at the end of each step was correlated to the vector of the previous step, thus a directional bias was incorporated into the pathway by setting the mean turning angle (*μ*) to 0, but variation in *μ* was drawn from a Gaussian distribution of turning angles with standard deviation (*σ*) (Bovet & Benhamou, 1988). As the pathway directional bias for the initial direction decreases over time, simulated pathways look relatively straight over short distances but show non‐unidirectional movements on a larger scale.

There are several pathway assumptions made in execution of these simulations. The number of steps in each simulated pathway was limited to a maximum. Thus, the maximum possible pathway length was the same for all pathways with the same fixed step‐length. The starting location for the simulations for each lake was set to real or simulated points at which the inflowing river discharged into the lake. The vector of the initial pathway was set to reflect the bearing at which that inflowing river intercepted the lake. The termination of a successful simulated migration pathway was defined in models as when the pathway crossed into an endpoint polygon close to the outlet (real or simulated) of the lake. Pathways that crossed into the endpoint polygon were defined as “successful migrations” whereas those that did not do so within the constraints of the maximum path length set for the model were defined as “unsuccessful migrations.” Where a path intersected with the lake boundary, a new pathway bearing was set, drawn from a distribution of turning angles with a slightly enlarged standard deviation (SD), for only this particular step, according to the equation:

$$\text{turning angle}\;\sigma =\sigma \times \left(1+0.1\times k^{2}\right)$$

where *σ* is the standard deviation of the turning angles and *k* = 0 initially, but *k* = (*k* + 1) for each subsequent pathway event that intersected with a lake boundary. This resulted in a higher turning angle standard deviation as the number of failed attempts increases, reducing the probability of constant collisions between pathways and lake boundaries.

### 2.2 Step‐length and turning angle distribution

Hanssen (2020) found that a pathway with a simulated step‐length of 50 m best fitted the empirical telemetry data for Atlantic salmon migrating through a Norwegian lake. Thorpe and colleagues (Thorpe et al., 1981) showed Atlantic salmon smolts migrating through Loch Voil exhibited mean step‐lengths ranging from 141 to 200 m. Thus, in this study we examined the effects on pathway characteristics of five simulated step‐lengths: 50, 75, 100, 150, and 200 m. To compare pathways, the number of simulated steps was reduced with increasing step‐length to give a maximum overall pathway length of 75 km across all simulations; a distance of at least eight times the direct travel distance for any of the simulated minimum migration distances.

Similarly, we tested the effect on simulated pathways all with a mean of angle of 0° but with four different turning angle distributions, drawn from a Gaussian distribution of turning angles, but with standard deviations (*σ*) of 2**°**, 5**°**, 15**°**, and 25**°**. To test the effect of step‐length and turning angle distribution on path characteristics, 200 pathways were simulated for all combinations of the four different turning angle distributions and five step‐lengths. Each simulation was run with an unbounded two‐dimensional space within the simulated lake margins, in R using the crw_in_polygon() function.

#### 2.2.1 Lake shape

**FIGURE 1:**
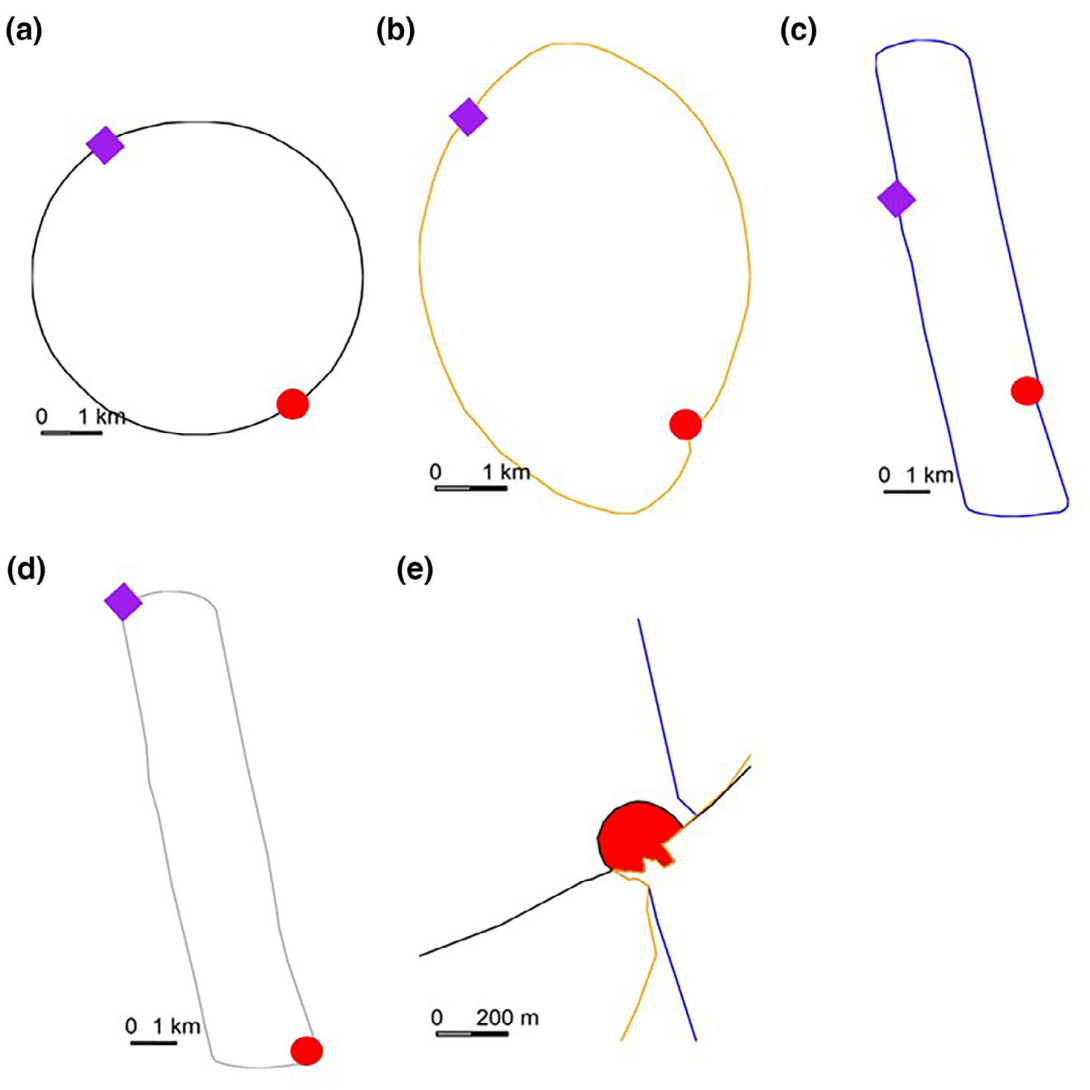
The four simulated lake shapes to which random walk model simulations were applied: (a) circular lake basin shape (area 25.0 km^2^), (b) elliptical basin shape (area 22.1 km^2^), (c) rounded‐rectangular basin (area 27.8 km^2^), and (d) terminal‐rectangular basin (area: 27.8 km^2^). The purple diamond is the start point for the simulated migrations. The area of each lake is the same and the angular coordinates for the starting location, endpoint position, and polygon size are identical for lake shapes (a), (b), and (c). For the terminal‐rectangular basin shape, the start point (purple diamond) and end point (red circle) are positioned at each end of the basin to better represent the most likely drainage pattern of real lakes of this shape. (e) Shows detail of the end point, a polygon of 440 m circumference. A simulated migration ends where the pathway crosses into this polygon.

To test the effect of basin shape on simulated migration pathways, three contrasting hypothetical lake shapes and two variants of one lake shape were created in QGIS 3.18.2 (QGIS Development Team, 2021). The lake basin shapes were circular, elliptical, and rounded‐rectangular (henceforth rectangular) (Figure 1). Each simulated lake was constructed with the same area (24.3 km^2^ ± 0.2%). The starting and end points of the simulated migration were set at similar positions on the circumference of each lake type and at an identical distance (5.5 km) apart. The migration end point for each simulated lake migration was identical and comprised a circular polygon with a 440‐m exposed perimeter (Figure 1). For the rectangular basin shape, the start and end points do not well reflect the glaciated fjord lakes that are most likely to exhibit such basin shapes. Thus, a second variant of the rectangular basin shape (the terminal‐rectangular shape; Figure 1) was created with migration start and end points reflecting afferent and efferent rivers at the ends of the basin. This had the effect of increasing the distance between the migration start and end points to 11.3 km, all other characteristics remaining the same. A simulation colliding with the end point was deemed to be a successful simulated migration.

For each set of simulations, a number of pathway characteristics were determined. “Migration success rate” was measured as the number of pathways that reached the end point as a proportion (%) of all simulations. “Distance travelled” (km) was the distance covered during a successful simulated path, calculated by multiplying the step‐length by the total number of steps taken to reach the end point. Lake passage time was defined as the time difference from the start of the lake migration of a real or simulated smolt to the end point.

#### 2.2.2 Comparing simulated and empirical migration pathways

To compare migration pathway characteristics (success rate and passage time) derived from random walk simulations with characteristics of actual salmon smolt migrations through real lakes, migration simulations were made for five lakes of differing shapes and sizes for which there are empirical smolt migration pathway data (Table 1). Four of these lakes (Lochs Achonachie, Meig, Garve, and Lomond) are in Scotland and three of them (Achonachie, Meig, and Garve) form part of the Conon catchment. The other lake (Bassenthwaite Lake) is in England. For each simulation of migration through the natural lakes, the actual basin shape and size was used. The starting point was the river of origin for which empirical Atlantic salmon smolt tracking data were available and the end point for simulations was the efferent river leaving the lake.

**TABLE 1:** The location of the five UK lakes for which random walk models were constructed and for which there are empirical lake migration data for salmon smolts.

Lake	Latitude; longitude	Surface area (km^2^)
Loch Achonachie	57°33.38′ N; 4°36.78′ W	0.69
Loch Meig	57°33.72′ N; 4°44.59′ W	0.45
Loch Garve	57°35.94′ N; 4°39.76′ W	1.83
Loch Lomond	56°06.61′ N; 4°37.16′ W	71
Bassenthwaite Lake	54 39.06′ N; 4°37.16′ W	5.1

Empirical data were derived from studies conducted on Atlantic salmon smolts migrating downstream in the afferent river and through each lake. Fish were tracked using acoustic telemetry. V7 coded 69 kHz transmitter tags (for Achonachie, Meig and Garve: tag size 7.3 mm diameter, 17 mm length, 1.8 g mass in air, Thelma Biotel; for Bassenthwaite and Lomond: 7 mm diameter, 19.5 mm length 1.5 g mass in air, InnovaSea Ltd) were inserted intra‐abdominally to smolts captured by rotary screw trap in each of the afferent rivers. Only Atlantic salmon smolts >130 mm fork length and >20 g weight were tagged. An assumption of the study presented here is that the very minor differences in tag size had no appreciable differential effects on fish migration success. Fish were detected using fixed position Vemco VR2W receivers (InnovaSea Ltd) located at the end of the afferent rivers as they entered the lake and at the mouth of the efferent river, to record fish entering and exiting the lake, respectively. In‐lake receivers provided additional data on fish position during lake passage and from this more detail on migration pathway distance was derived. Data on smolt migration characteristics for Loch Lomond are provided in more detail in Lilly et al. (2022) and for Lochs Achonachie, Meig, and Garve in Honkanen et al. (2021).

**FIGURE 2:**
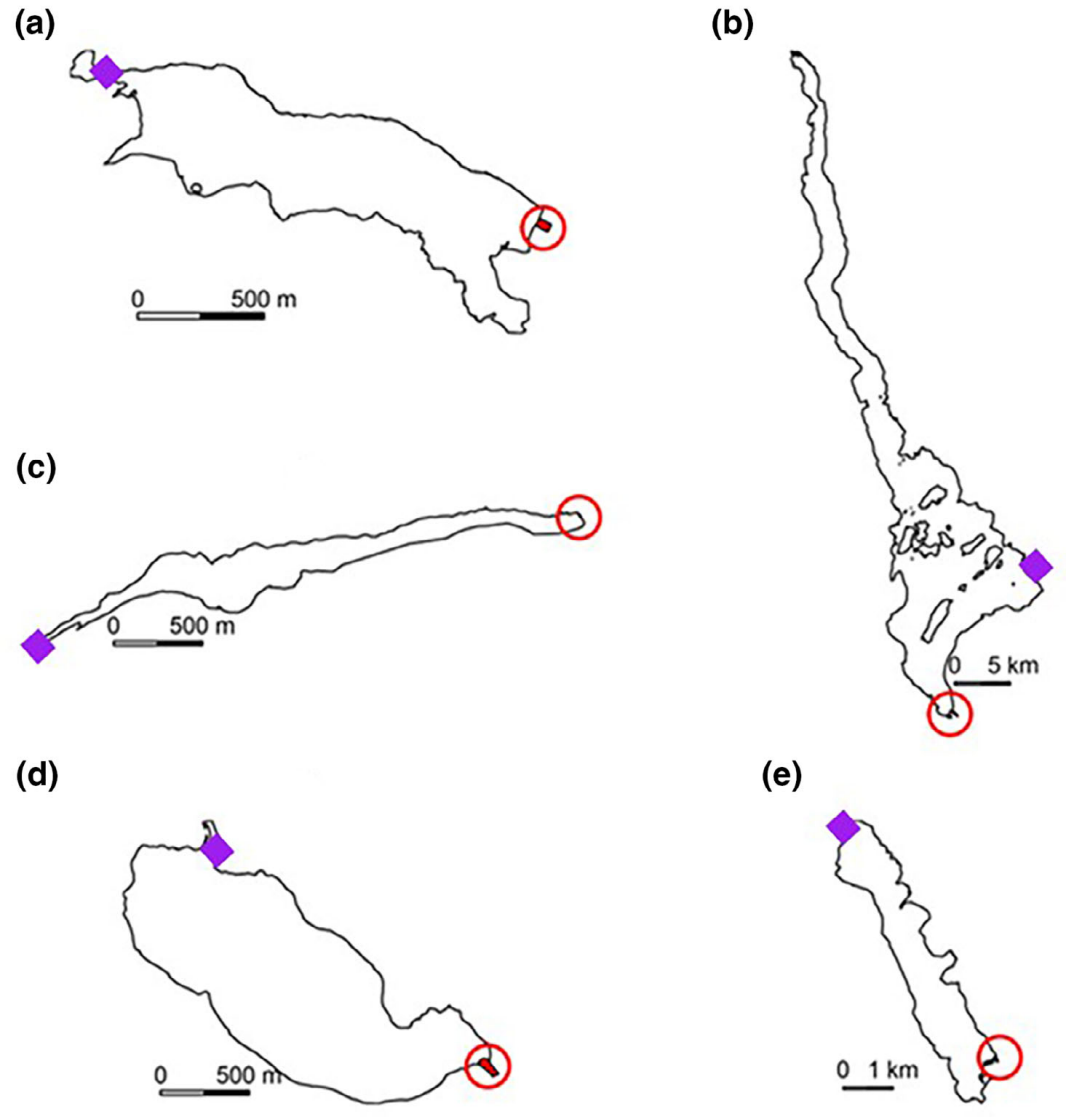
The five lakes used to compare empirical telemetry data with simulations of migration: (a) Loch Achonachie (surface area 0.69 km^2^), (b) Loch Garve (surface area 1.83 km^2^), (c) Loch Meig (surface area 0.45 km^2^), (d) Bassenthwaite Lake (surface area 5.1 km^2^), and (e) Loch Lomond (surface area 71 km^2^). Purple diamonds indicate the starting position of the pathway simulations and the red circles mark the migration end points for each lake.

The start point of simulated smolt migrations was set to the position of the receiver at the mouth of the afferent river and the initial movement vector was set to match the direction of the intercept of the inflowing river to each lake. The end point was located to cover the opening of the efferent river of each lake except for Loch Meig, which is impounded and fish exit the lake by means of a Borland fish lift. Here the end point was an endpoint polygon around the fish lift itself. As the shape and size of the mouth of the rivers draining each lake differed, to attempt to make the simulated migrations match as closely as possible the real migration, the simulated endpoint size differed for each lake. Thus the endpoint polygons for the five lakes had exposed circumferences of 31 m for Achonachie, 50 m for Garve, 29 m for Meig, 73 m for Bassenthwaite, and 200 m for Lomond (Figure 2).

Simulations of passage through real lakes were conducted for three migration pathway step‐lengths (50, 100, and 200 m) and for three *σ* values of turning angle distribution (5°, 15°, and 25°). For each combination of step‐length and turning angle distribution, 200 simulations were made, resulting in a total of 1800 individual simulated pathways for each lake. The maximum allowable migration pathway length for each simulation could vary between lakes. Maximum allowable simulated migration pathway length was adjusted between simulations on different lakes depending on the empirical acoustic telemetry data. The longest successful migration times for each lake were 5.58 days (Bassenthwaite), 21.79 days (Lomond), 30.21 days (Garve), 31.71 days (Meig), and 34.32 days (Achonachie). For each lake, the maximum simulated migration distance was calculated using a common swim speed of 0.17 m s^−1^. The maximum allowable pathway length for simulations on each lake was determined as the product of swim speed (0.17 m s^−1^) and the longest time taken for a successful migration was determined from the empirical telemetry data.

To successfully run the simulations, the maximum allowable pathway lengths for simulated migrations in Lochs Lomond, Achonachie, and Meig were constrained to a maximum of 75 km. As a result of the quicker migration times and shorter migration distances of the smolts tracked by telemetry, the maximum allowable pathway lengths of migration simulations in Loch Garve and Bassenthwaite Lake were 55.8 and 30.4 km, respectively.

To calculate a measure of lake passage time for each successful simulated pathway, the distance travelled was divided by the mean movement speed of smolts recorded across all five lakes, which was 0.17 m s^−1^.

### 2.3 Ethics statement

The use of animals in this study complied with the UK Animal (Scientific Procedures) Act 1986 and was conducted under UK Home Office license number PP0483054.

### 2.4 Statistical analyses

A generalized linear model (GLM) with a binomial probability distribution was used to test whether the categorical variable, lake shape (four levels), or continuous variables step‐length and turning angle standard deviation (*σ*) and their interactions influenced the success of simulated smolts. Model selection and variable significance were based on likelihood ratio tests between nested models. A final model was selected when no further variables could be removed without reducing the significance of the model. Model diagnostics were performed using the plotSimulatedResiduals function from the DHARMa package in R (Hartig, 2020) and through residual plots. Odds ratios for variables influencing the success rate across different lake shapes were calculated from GLM model outputs using the glm function in the oddsratio package in R (Schratz, 2017).

Lastly, chi‐squared tests were performed to assess whether there were any significant differences in the frequency of successful and unsuccessful migrants at varying step‐lengths and turning angle *σ* values. Where normality could not be confirmed with Shapiro–Wilks tests and residual plots, the Kruskal–Wallis rank sum test was used followed by Dunn's Kruskal–Wallis multiple comparisons test. All *p* values reported are after Holm–Bonferroni correction. All analyses were conducted within R V4.0.3 (R Core Team, 2020).

## 3 RESULTS

### 3.1 Migration success

**FIGURE 3:**
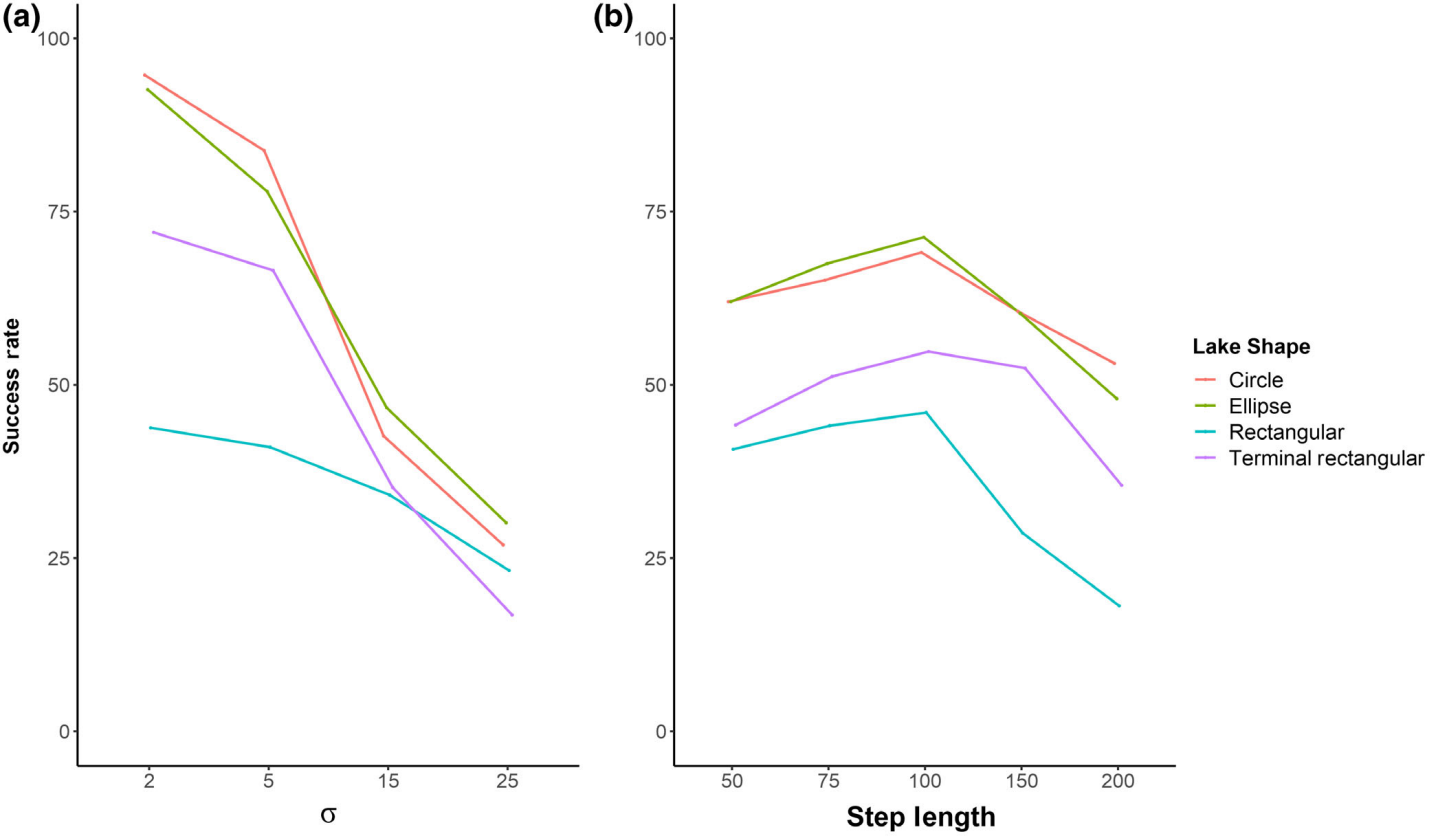
(a) The success rate (%) of simulated migrations for turning angles with a mean of 0° drawn from a Gaussian distribution of angles with varying standard deviation (*σ*) values (2°, 5°, 15°, and 25°) Each group comprised 200 individual simulations of each step‐length (50, 75, 100, 150, and 200 m) for each of the four different basin shapes. (b) The success rate (%) of simulated migrations for five different step‐lengths (50, 75, 100, 150, and 200 m across the four different basin shapes or start/endpoint combinations. Each group comprised 200 simulations of each turning angle distribution and step‐length.

There was a significant general trend in simulated migrations for all lake shapes, where migration success declined with increasing variation in turning angle distribution (*σ*) (odds ratio 0.842, 97.5% CI 0.823–0.861) (Figure 3a). There was also a significant effect of step‐length on migration success (odds ratio 0.993, 97.5% CI 0.991–0.996). Increasing step‐length resulted in increased migration success up to a step‐length of ca. 100 m, after which migration success rate declined markedly (Figure 3b).

Migration success rate for simulated migrations was dependent on lake shape, step‐length, and the turning angle distribution (*σ*) of the simulated pathway. Modelling showed a significant three‐way interaction between lake shape, step‐length, and turning angle *σ* (2Δ log‐liklihood = 9.13, *df* = 3, *p* = 0.028). Across all simulations, circular and elliptical basin shapes had higher migration success rates in comparison to the rectangular basin shape (Figure 1), irrespective of pathway characteristics (Figure 3a,b). In simulations where the basin shape changed but the distance between migration start and end points did not (Figure 1a–c) simulated migration success was around 49% greater for the circular and elliptical lake shapes than for the rectangular lake shape for a turning angle distribution *σ* of 2°, reducing to around 6% for *σ* = 25° (Figure 3a). Similarly, the simulated migration success for circular and elliptical lake basin shapes was ca. 22% and 32% greater than for the rectangular basin shape for models with step‐lengths of 50–200 m, respectively, for lake combinations with the same distance between model start and end points (Figure 3b). For the two modelled scenarios, where basin shape and size remained constant but the simulated start and end points were either lateral or terminal on the lake (rectangular and terminal‐rectangular in Figure 1), the modelled migration success rate was generally greater where the migration start and end points were terminal as opposed to lateral on the lake. Thus, the rectangular basin shape returned a much lower probability of migration success (odds ratio 0.128, 97.5% CI 0.082–0.200) than the terminal‐rectangular lake shape (odds ratio 0.347, 97.5% CI 0.218–0.551). The effect of increasing turning angle variation (*σ*) in simulations of the two rectangular lakes showed differing degrees of decline in success rate. The migration success rate in the rectangular lake shape with terminal start/end points showed a steeper decline overall (odds ratio 0.999, 97.5% CI 0.969–1.031) compared to the lateral start/end points (odds ratio 1.101, 97.5% CI 1.070–1.134). Thus, simulated migrations through a rectangular lake shape with terminal start/end points were 27% more successful in simulations with turning angle distributions of *σ* = 2°, although marginally (4%) less successful at *σ* = 25°. For step‐lengths of 50 m, simulated migrations through the rectangular lake shape with terminal start/end points did not differ (4%), but they were significantly (18%) more successful with step‐lengths of 200 m (odds ratio 0.995, 97.5% CI 0.991–0.998).

### 3.2 Distance travelled

**FIGURE 4:**
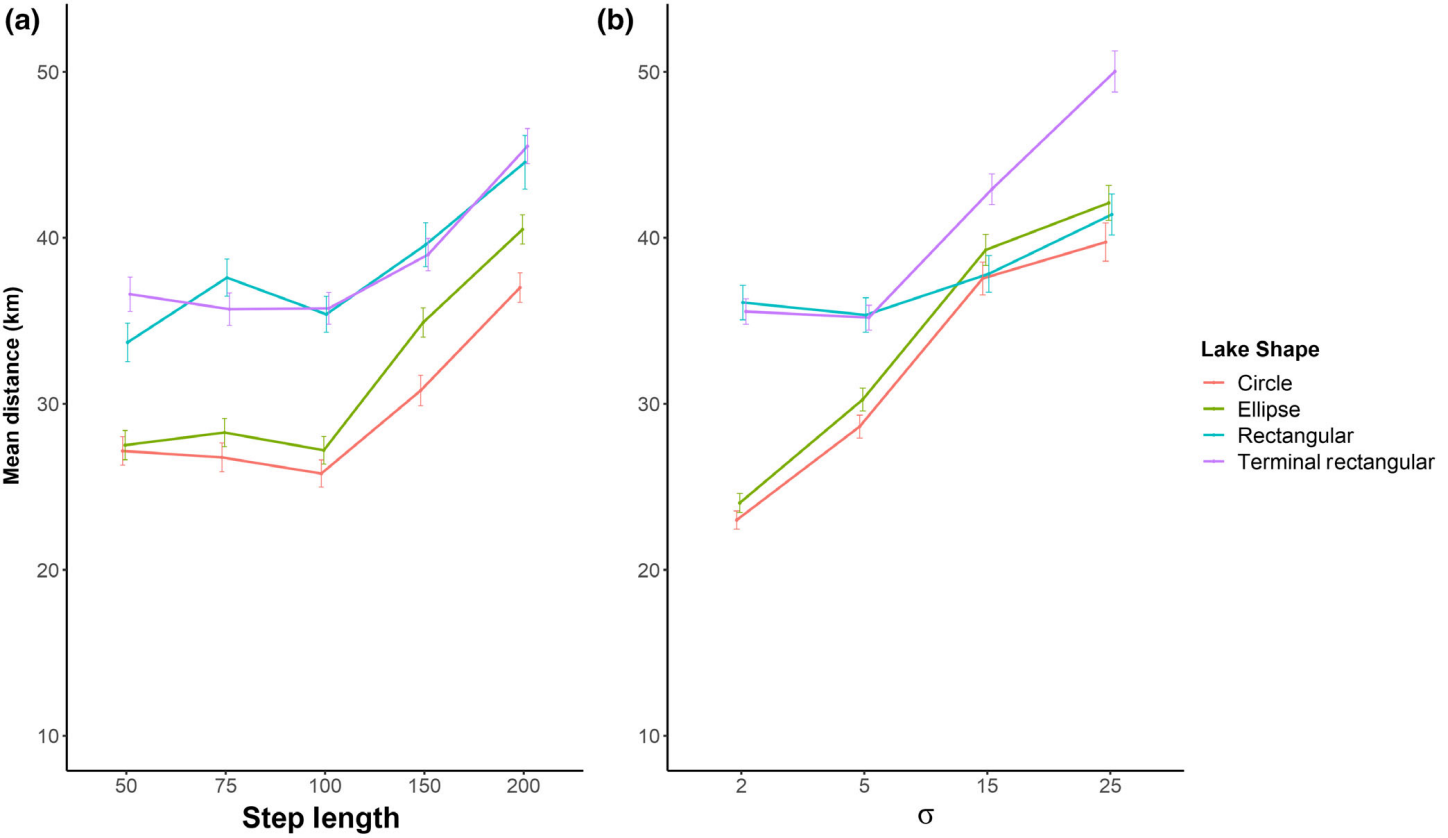
(a) The mean distance travelled (with standard errors) by simulated fish making a successful migration on pathways with varying step‐lengths (50, 75, 100, 150, and 200 m) across the four different basin shapes, each comprising 200 simulations of each of four turning angle *σ* values of 2°, 5°, 15°, and 25°. (b) The mean distance travelled (with standard errors) by simulated fish making a successful migration on pathways with a turning angle with a mean of 0 but drawn from a Gaussian distribution with four standard deviation (*σ*) values (2°, 5°, 15°, and 25°) across the four different basin shapes. Each plot comprises the mean of 200 simulations of each of five step‐lengths, 50, 75, 100, 150, and 200 m.

Mean distance travelled by fish making successful migrations varied with lake shape (Figure 4). For both circular and elliptical lake shapes, the mean distance travelled was shorter than for the rectangular lake shape, the effect being particularly pronounced when variation in turning angle distribution was low (ca 35% shorter at *σ* = 2 but only 0.4% at *σ* = 25) (Figure 4a). Similarly, for round and elliptical lake shapes, the mean distance travelled was shorter than for the rectangular lake shape over all step‐lengths, for example 17% and 8% shorter (for circular and elliptical lake shapes, respectively) at a step‐length of 200 m. The effect was particularly pronounced when step‐length was relatively small (at 50 m) when the distance travelled was ca. 18% shorter (Figure 4b). Migration distances were generally greater for simulated migrations in the rectangular lake with terminal start/end points than for the same lake with lateral start/end points but that effect was dependent on turning angle distribution, the effect becoming larger with greater variation in turning angle. Thus, at a turning angle distribution (*σ*) of 2°, mean migration distance for both lake types was within 2%, but at *σ* = 25° the distance travelled by fish in the terminal start/stop point lake was 18% longer (Figure 4a). The effect of step‐length on the travel distance difference between lateral start/end point and terminal start/end points was small, with the terminal start/end point condition distance travelled being 8% more than the alternative for a step‐length of 50 m and 19% for a step‐length of 200 m (Figure 4b).

**FIGURE 5:**
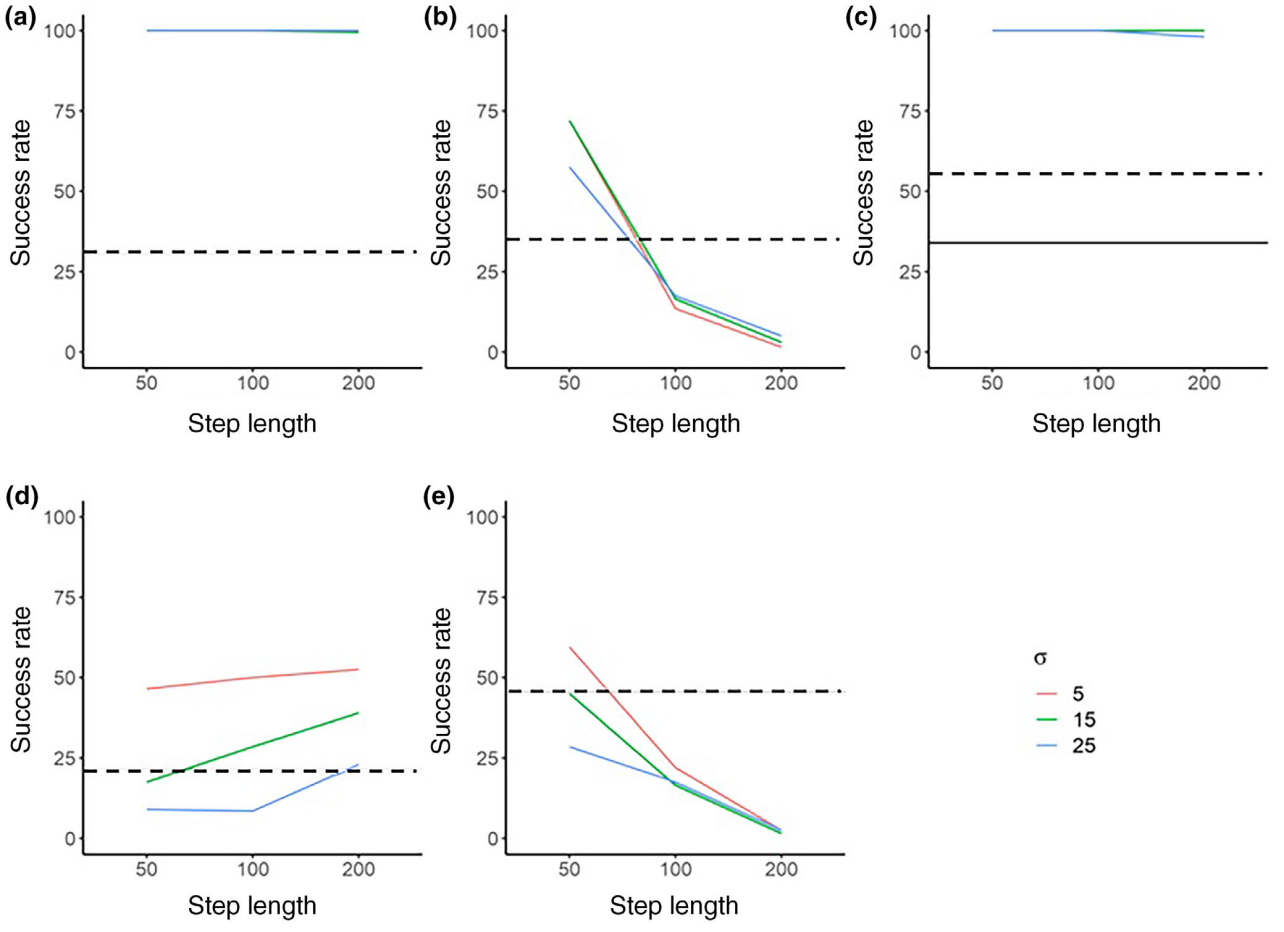
The migration success rate determined from 200 simulations for mean turning angles of 0° but drawn from a Gaussian distribution of turning angles with three standard deviation (*σ*) values (5°, 15°, and 25°) and three step‐length values (50, 100, and 200 m) across the five different lakes: (a) Loch Achonachie, (b) Loch Meig, (c) Loch Garve, (d) Loch Lomond, and (e) Bassenthwaite Lake. The dotted horizontal lines show the observed migration success rate through each lake for salmon smolts tagged with acoustic telemetry transmitters. The solid line shows success rate from fish tagged with PIT tags. The telemetry derived observed migration success rate of smolts through Loch Achonachie was 31% (*n* = 5), Loch Meig 35% (*n* = 6), Loch Garve 34% PIT tagged fish (solid black line) and 55% acoustic tagged fish (*n* = 12), Loch Lomond 21% (*n* = 28), and Bassenthwaite Lake 46% (*n* = 15).

The pathways simulated for migration though Lochs Achonachie and Garve had the highest rates of success (Table 2; Figure 5). For these lakes neither turning angle distribution nor step length had an appreciable effect on migration success rate, which was 100% or close to this for all permutations. For both lakes the simulated migration success rate was markedly higher than the empirical migration success of tagged smolts (31% and 34% for Lochs Achonachie and Garve, respectively).

**TABLE 2:** The empirical measures of smolt migration success and the model parameters (step‐length and turning angle *σ*) applied to a random walk model that result in similar migration success outcomes.

		Model parameters matching empirical migration success					
Lake	Empirical migration success (%)	Step‐length	*σ*	Step‐length	*σ*	Step‐length	*σ*
Achonachie	31	None	None				
Meig	35	99	5	101.7	15	82.4	25
Garve	55	None	None				
Lomond	21	74.7	15	190.7	25		
Bassenthwaite	46	76.4	5	47.4	15		

For the three lakes from the same catchment (Lochs Achonachie, Meig, and Garve) there was a significant between‐lake difference in the success of simulated migrations across all three step lengths: 50 m, *χ* ^2^ = 442.42, *df* = 2, *p* < 0.001; 100 m, *χ* ^2^ = 1403.9, *df* = 2, *p* < 0.001; 200 m, *χ* ^2^ = 1693.6, *df* = 2, *p* < 0.001 (Figure 5a–c).

For simulations of fish migration through Meig, Bassenthwaite, and Lomond, simulated migration success rates were more in line with those determined from empirical study, with at least one combination of modelled step length or turning angle *σ* intersecting with the empirically determined success rate (Figure 5b,d,e).

There was a significant interaction effect between turning angle *σ* and step‐length (2Δ LL = 31.414, *df* = 1, *p* < 0.001) on migration success. Thus, the generally negative effect of increasing turning angle *σ* on success rate was lessened in the pathways with larger step lengths (odds ratio 1.001, 97.5% CI 1.000–1.001). Further significant interactions were also detected between turning angle *σ* and lake (2Δ LL = 104.87, *df* = 4, *p* < 0.001) and between lake and step‐length (2Δ LL = 675.32, *df* = 4, *p* < 0.001) such that the effects of both turning angle *σ* and step‐length varied depending on the lake.

For simulations of fish migration through Loch Meig and Bassenthwaite Lake, success rate declined with increasing step‐length; in contrast, success rate increased with increasing step‐length for fish migration simulations through Loch Lomond. For Lochs Meig and Lomond, and Bassenthwaite Lake, migration success generally declined with increasing variation in pathway turning angle distribution (*σ*). However, this effect was most pronounced in Lomond (odds ratio 0.94, 97.5% CI 0.702–1.272), the effect was smaller in Bassenthwaite (odds ratio 1.022, 97.5% CI 0.759–1.376), and very considerably reduced in Meig (odds ratio 1.059, 97.5% CI 0.786–1.426).

Simulated migration success rates exceeded that of tagged fish migration through Loch Meig (35%) when pathway step‐length was short (less than 75 m) for any turningangle distribution. There were no significant differences between the success rate of fish tracked using telemetry and simulated migrations with step‐lengths of 100 or 200 m for any turning angle distributions (*p* ≤ 0.14). In Bassenthwaite Lake, empirically measured migration success (46%) was only exceeded by simulations when the step‐length was less than 75 m and the turning angle distribution was 5° or less. Modelled conditions comprising a step‐length of 50 m and a turningangle distribution of 15° also closely predicted empirically measured migration success. Migration simulations through Loch Lomond showed a different pattern from that of Loch Meig and Bassenthwaite Lake. Although modelled migration success generally increased with narrower distributions of turning angle, increasing step‐length generally increased the migration success rate (odds ratio 1.148, 97.5% CI 0.72–1.272). Thus, the simulated migration success exceeded that of the empirical migration success (21%) for all modelled step‐lengths when turning angle *σ* = 5°, when step‐length was greater than 60 m for a *σ* = 15°, and when step‐length exceeded 200 m for the highest turningangle distribution (*σ* = 25°). The closest simulation results showing success rates similar to those of the telemetry data resulting from combinations of 50 m step‐lengths with a turning angle *σ* = 15° (success rate = 17.5%, *n* = 35) and simulations consisting of 200 m step‐lengths with a *σ* = 25° (success rate = 23%, *n* = 46).

### 3.3 Simulated migration passage times for real lakes

**FIGURE 6:**
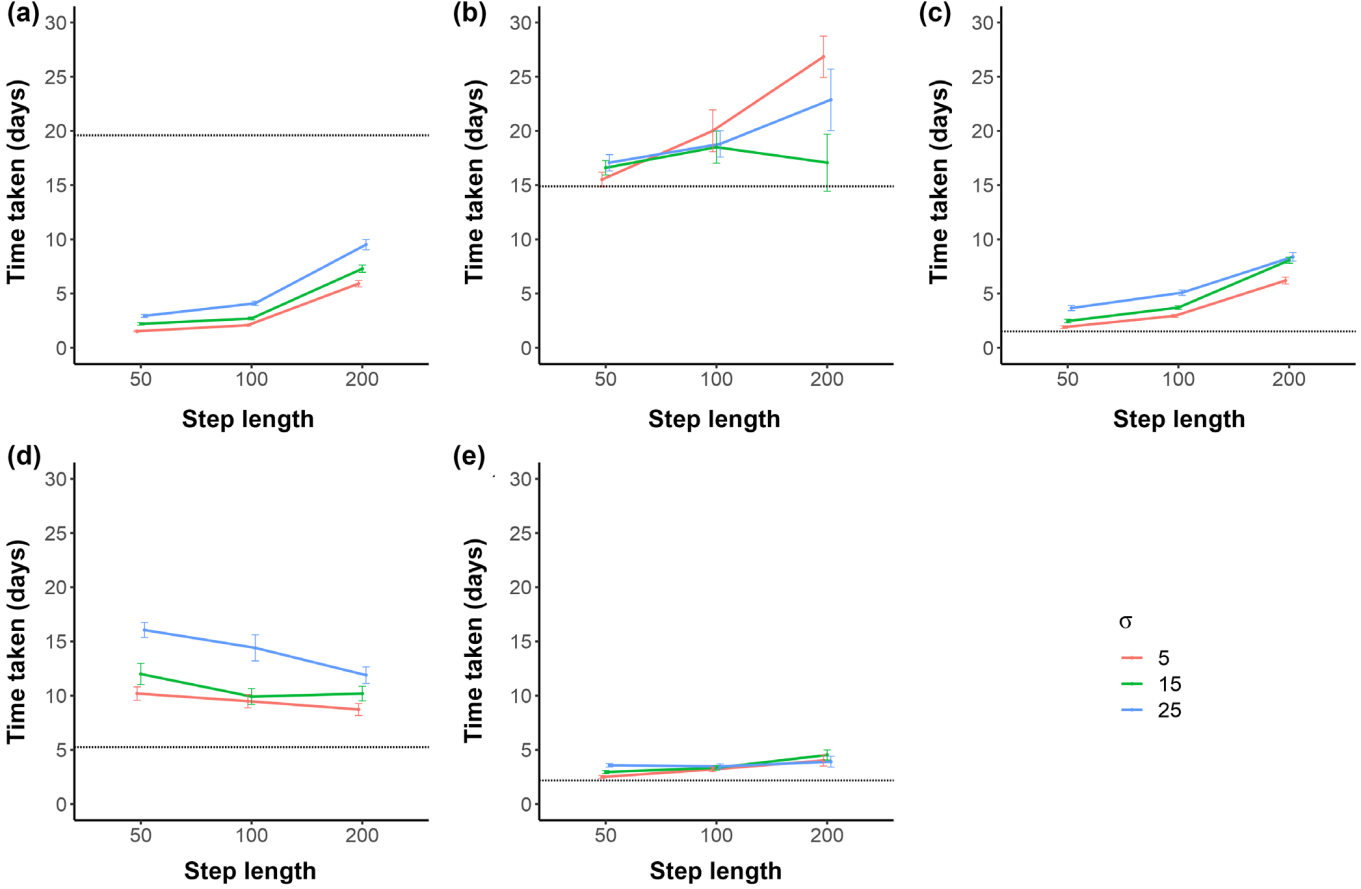
The mean travel time (±SD) for successful simulated migrations modelled with turning angles of mean = 0 but drawn from a Gaussian distribution with three standard deviation (*σ*) values (5°, 15°, and 25°) and three step‐length values (50, 100, and 200 m) for each of the five lakes: (a) Loch Achonachie, (b) Loch Meig, (c) Loch Garve, (d) Loch Lomond, and (e) Bassenthwaite Lake. The mean travel times assume that simulated fish are migrating continuously for 24 h each migration day. Dotted horizontal lines show the observed mean travel time of the successfully migrating smolts determined from acoustic tag telemetry data. Empirically measured mean and SD for migration durations of smolts through Loch Achonachie were 19.6 ± 7.6 days, Loch Meig 14.9 ± 17.5 days, Loch Garve 1.5 ± 1.4 days, Loch Lomond 5.2 ± 4.2 days, and Bassenthwaite Lake (2.2 ± 1.4 days).

The model simulated migration time for lake passage was markedly shorter (indicating a faster passage) than the measured mean migration times derived from the empirical study for Loch Achonachie (Figure 6a) for all permutations of step‐length and turning angle distribution modelled. In contrast, all model permutations for the other four lakes indicated a slower passage time for simulated smolts than the empirically measured passage time (Table 3).

**TABLE 3:** The migration passage times in days for each of five lakes measured empirically by telemetry and the range (slowest and fastest) of lake passage migration times and the step‐length (m) and turning angle *σ* parameters delivering that passage time, derived from random walk modelling (Figure 4).

		Slowest modelled passage			Fastest modelled passage		
Lake	Empirical passage time (mean) (days)	Time (days)	Step‐length	*σ*	Fastest modelled passage (days)	Step‐length	*σ*
Achonachie	19.6	**9.6**	**200**	25	1.8	50	5
Meig	14.9	26.8	200	5	**15.7**	**50**	**5**
Garve	1.5	8.2	200	25	**2**	**50**	**5**
Lomond	5.2	16.1	50	25	**8.4**	**200**	**5**
Bassenthwaite	2.2	4.8	200	15	**2.6**	**50**	**5**

*Note*: Parameters in bold result from the model construction that most closely matches empirical measures of passage time.

For three of these four lakes, the random walk parameters that most closely predicted the empirically measured migration passage time was the shortest step‐length (50 m) and the least variable turning angle distribution (*σ* = 5°). For the fourth lake (Loch Lomond) the model parameters that best predicted migration passage time was also the least variable turning angle distribution but, in contrast, the longest step‐length modelled.

Only simulated pathways with step‐lengths of 100 m showed a significant difference between all three lakes (Kruskal–Wallis *χ* ^2^ = 265.93, *df* = 2, *p* < 0.001) with a Dunn's test revealing each loch was significantly different from one another (Meig–Achon: *Z* = −16.19, *p* < 0.001; Garve–Meig: *Z* = −12.99, *p* < 0.001; Achonachie–Garve: *Z* = −6.12, *p* < 0.001). At the remaining step‐lengths of 50 and 200 m, while both Achonachie and Garve showed significant differences in migration time to Meig (50 m: Kruskal–Wallis *χ* ^2^ = 803.14, *df* = 2, *p* < 0.001; 200 m: Kruskal–Wallis *χ* ^2^ = −39.08, *df* = 2, *p* < 0.001), they did not differ significantly between one another (Achon–Garve 50 m: *Z* = −0.56, *p* = 0.579; 200 m: *Z* = 0.19, *p* = 0.85).

## 4 DISCUSSION

Migration by Atlantic salmon from freshwater breeding and juvenile nursery areas to marine feeding grounds smolts is clearly a high‐risk behavior. The magnitude of that risk, however, differs between rivers and between the habitat types through which salmon smolts migrate (Thorstad et al., 2012). There is now robust evidence that salmon smolt migration success through standing bodies of water (lakes and reservoirs, but potentially other standing waters also) is generally very low in comparison to migration through other habitat types, with success rates in the range of only 10%–60% commonly reported (Aarestrup et al., 1999; Honkanen et al., 2018; Kennedy et al., 2018; Lilly et al., 2022; Mclennan et al., 2018; Thorpe et al., 1981). Tracking studies of smolt behavior during migration in standing waters shows consistent evidence of seemingly chaotic, random pathways that frequently take fish in directions away from the efferent river and thus markedly extend their total migration distance and time (Honkanen et al., 2018; Lilly et al., 2022). For example, the mean pathway length of Atlantic salmon smolts successfully migrating through Loch Lomond in one study was in excess of 56 km, taking on average 5.2 days, with one fish travelling over 246 km and taking 22 days. Thus, these smolts travelled on average 6.4 times further (but up to 28 times further) than the most direct route through the lake (8.8 km) (Honkanen et al., 2018; Lilly et al., 2022). Slower migration rates are known to lead to increased risk of migration failure, in part through an elevated risk of predation (Huusko et al., 2017).

A logical interpretation of these consistent observations of migration through standing waters is that the cues required for navigation are lacking (Lennox et al., 2021; Thorpe et al., 1981). When animals lack information on the position of their target destination it is frequently assumed that they use some form of random search strategy to reach it (Bartumeus et al., 2005). Hanssen (2020) showed that the pattern of movement by Atlantic salmon smolts in a Norwegian lake (Evangervatnet) more closely resembled random search movements than directed navigation. Consistent with the results of Hanssen (2020), Lilly et al. (2022) noted that salmon smolts migrating through Loch Lomond displayed behavior similar to simulated smolts up until they reached an area around the outflowing river. Once in this zone, a high proportion of smolts successfully migrated out of the lake. These authors speculated that this was because directional cues from water currents created by the outflowing river became apparent to the migrants on entering this zone. They called this zone the “Goldilocks Zone” because here the migration conditions were “just right” (Cundall, 1849).

In the study presented here, we tested the prediction that a correlated randomized walk search strategy for smolts migrating though a standing water body was affected by the shape of the lake basin. The modelling results presented here clearly show that fish adopting a navigation strategy based on a random walk were more successful in migrating through a lake with a round or elliptical basin shape in comparison to a rectangular shape. Success in this context was measured as successful migration to a predetermined end point. However, some simulated fish that were successful migrants travelled up to 75 km to cover a 5.5‐km distance between lake entry and exit points. In addition, for rectangular lakes with a basin shape similar to that of a glacial ribbon lake, the location of the migration start and end points had a significant effect on migration success. For simulated lakes of the same size, but with migration start and end points either lateral or terminal to the lake (Figure 1c,d), modelling showed that migration success was markedly greater for the lakes with terminal start and end points under almost all modelled circumstances despite the minimum migration distance being greater in the latter lake type (Figure 3a,b).

For all modelled conditions, the total distance travelled by simulated fish using a randomized search strategy markedly exceeded the minimum migration distance (5.5 km) by a factor of between 4 and 10 (depending on model parameters) (Figure 4a,b).

Varying two of the principal components of the random walk model provided us with an insight into the detail of the behavioral tactics that are most likely to result in successful migration for fish adopting random search behavior. In general, a lower variation in the distribution of turning angles (a low turning angle *σ*) was more likely to result in migration success than for random walk pathways with high variation in turning angle (a high turning angle σ). Thus, passage success was 65% and 66% higher for random walk pathways with a turning angle *σ* = 2° than for *σ* = 25°. Step‐length also had a marked effect on migration success, showing a similar pattern amongst all lake shapes tested. However, there is some evidence that an intermediate step‐length was more likely to result in the highest migration success. Thus step‐lengths of around 100 m returned migration success rates higher than both smaller and larger step‐length for all lakes of the shape, size, and minimum migration distance tested in this study (Figure 3b). For example, migration success was 70% and 72% for circular and elliptical basin shapes at a step‐length of 100 m compared to 62% and 62% at a step‐length of 50 m, and 53% and 48% at a step‐length of 200 m, respectively.

These findings point towards a clear migration strategy for smolts migrating through a lake where navigational cues are insufficient to guide pathway. At least for small to medium sized lakes, a random search strategy can result in a successful migration. Such a navigation strategy will have greater success in lakes with rounded basin shapes, compared with long thin rectangular basins. Amongst rectangular shaped lakes, a random search strategy is more likely to be successful when the lake migration starting and end points are at alternative ends of the lake.

In the study presented here, we also compared the characteristics of multiple correlated random walk simulations where two key parameters of the random walk (step‐length and turning angle distribution) were varied against the characteristics of actual Atlantic salmon smolts tracked by telemetry though each of five different lakes in the UK. For two lakes, Lochs Achonachie and Garve, the simulated migration success for all model parameter conditions vastly exceeded that of real fish by a considerable margin (Figure 5a,c). In contrast, for Loch Meig, Loch Lomond, and Bassenthwaite Lake, the correlated random walk simulations predicted the actual migration success rate measured for salmon smolts tracked through the lake. There was a tendency for simulated and actual migration success rates to coincide when step‐lengths varied between 50 and 100 m (with one exception, for longer step lengths in Loch Lomond) and for smaller turning ‐angle distributions (*σ*) to be more successful than greater turning angle distributions. This coincides with the findings from random walks simulations on simulated lakes suggesting that at least for lakes of the types, size, and start/end points simulated here, a successful migration strategy in the absence of navigation cues would be a correlated random walk pattern comprising short step‐lengths and a relatively narrow variation in turn angle between steps.

In contrast to Loch Meig and Bassenthwaite Lake, simulated migration success rate increased with longer step‐lengths for simulated migrations through Loch Lomond. This lake is markedly different physically from the other four lakes modelled here. It is much larger (with a surface area of 71 km^2^; Bassenthwaite Lake is only 5.1 km^2^ and the remaining three lakes are less than 2 km^2^; Figure 2). In addition, the lake migration start point (the inflowing River Endrick in the southeast corner) and end point (the outflowing River Leven in the southwest corner) are both tucked into embayments in the lake and thus do not face each other, arguably presenting additional navigational migration difficulties for migrating salmon smolts. The output of simulations for migration through the much larger and complex Loch Lomond confirm those of Lilly et al. (2022), that a correlated random walk can be a successful navigation strategy for this migration. They also align with the finding from models for the four other lakes that a narrow distribution of variation in turning angle between migration steps results in a more successful migration outcome than a greater range of variation. However, for this larger, complex lake, simulations also indicate that a random search pattern typified by longer step‐lengths between direction changes also result in greater migration success. This contrasting finding suggests that the best random search migration strategy is not the same for all lake types and migration formats.

Migration success rates through Lochs Achonachie and Garve are not as easy to explain. Here it would appear that migrating smolts are not adopting a random search strategy or, if they are, then they are using movement parameters of turning angle and step‐length that are outside those modelled in this study. The evidence here is that were they to adopt a random search strategy, then their migration success would be higher than that indicated by empirical tracking studies. Thus, the question remains why did they not adopt such a migration tactic? One possibility is that for fish migrating in these two lakes, the cues used for navigation are present (Bartumeus et al., 2005). Salmon smolts migrating through rivers are widely thought to be rheotactic, responding to water flow as the principal directional cue (Hvidsten et al., 1995; Veselov et al., 1998). For fish migrating down river into a lake it is reasonable to assume that the flow cues may need to drop below some threshold for a change in migration tactics to switch from rheotaxis to a random search strategy. The evidence from this study is that for smolts migrating in these two lakes, the switch to a random search strategy did not occur. Ironically, if it had, then migration success is likely to have been higher than that shown from empirical measures of success. Another possible explanation is that random walk simulations for smolts in these lakes are over‐simplifications of either the performance of the fish and or the environment in which fish in these lakes are migrating. Larger fish generally have faster swim speeds (Wolter & Arlinghaus, 2003), but the size differences between smolts in different lakes were small (lake‐specific mean fork‐length ranging from 141.9 to 148.4 mm), thus differential fish size is highly unlikely to impact on variation in migration speed substantively. Lakes with short retention times are more likely to retain some directional currents and thus fish may be able to continue to use flow cues for navigation. The risk of predation has been shown to impact the pace of migration in the Chinook salmon, *Oncorhynchus tshawytscha* (Sabal et al., 2021). We have no concurrent data retention times or predation risk for smolts in the five lakes in this study, but it is certainly plausible that the risk of predation and/or lake retention time impact differentially on lake migration success in this study. Lastly it is possible that in some lakes smolts suspend migration to engage in lake feeding. There are no data in the literature of salmon smolts feeding in freshwater lakes during migration and the possible consequences of lake feeding on lake transit times, the pathways used, or ultimate lake migration success. There are thus a number of elements of lake migration by Atlantic salmon smolts worthy of further investigation.

The time of migration for smolt migrations in simulated random search passages through Loch Meig and Bassenthwaite Lake closely resemble the passage time of real smolts measured empirically, further strengthening the suggestion that this migration strategy is being used in these lakes. For Loch Lomond, the passage time for real smolts was markedly faster than for the simulated pathways. One possible explanation for this is provided by Lilly et al. (2022). This study showed that there is an area of the lake adjacent to the outflowing River Leven where smolts entering this area have a much higher probability of successfully migrating out of the lake than they do from other points in the lake. Lilly et al. (2022) speculate that this is an area of the lake (the Goldilocks zone) where cues to navigation become more apparent and thus fish may be switching from a random search strategy back to a cue‐directed navigation migration strategy again. The shorter passage times taken by actual Loch Lomond migrating fish, compared with the simulated random search migrations, might suggest that the Goldilocks zone is relatively large in Loch Lomond and indeed this has been estimated as extending to ca. 2 km from the outflowing River Leven (Lilly et al., 2022). The passage times for simulated migration through Loch Garve fairly closely match those of tracked smolts (Figure 6), suggesting that the navigation mechanisms that successful migrants use result in passage that is very similar to that of fish adopting a random search migration strategy, albeit that far fewer fish are successful lake migrants (Figure 5). Real smolts making successful migrations through Loch Achonachie were considerably slower than the time predicted by simulations of fish adopting a random search migration strategy (Figure 6) and there were fewer of them (Figure 5). This further indicates that, unlike in some of the other lakes examined here, smolts migrating thorough Loch Achonachie are not adopting a random search migration strategy and that the migration strategy that successful migrants do adopt results in a passage that is much slower than it would be if they did adopt such a strategy, or that smolts are suspending migration for a period of time.

We find that this study supports the conclusion that sea‐migrating salmon can switch from rheotaxis in rivers to a random search strategy to find the outflowing river in lakes and that this strategy can lead to successful egress of fish from the lake system. We speculate that this switch may not occur if water flow provides some directional cues but if these cues are not reliable then failing to switch to a random search strategy may be suboptimal. For small lakes a search strategy that comprises swimming in short straight lines with only small angular deviations between steps will be the most successful. For larger lakes, larger step lengths may be more optimal.

## AUTHOR CONTRIBUTIONS

MN: concept development, field data collection; model development, manuscript review and editing; JMcC: model execution, manuscript drafting and editing; HMM: concept development, field data collection; manuscript review and editing; AS: concept development, funding acquisition manuscript drafting and editing; JML: field data collection, manuscript review and editing; DLO: field data collection and analysis, manuscript review and editing; AG: field data collection, manuscript review and editing; LC: concept development, field data collection, manuscript review and editing; JRR: concept development, manuscript writing and editing; CEA: funding acquisition, concept development, data analysis, manuscript writing and editing.
